# Under what circumstances can immigrant patients and healthcare professionals co-produce health? - an interpretive scoping review

**DOI:** 10.1080/17482631.2020.1838052

**Published:** 2020-10-28

**Authors:** Christina Radl-Karimi, Anne Nicolaisen, Morten Sodemann, Paul Batalden, Christian von Plessen

**Affiliations:** a OPEN Odense Patient Data Explorative Network, University of Southern Denmark and Odense University Hospital, Odense C, Denmark; b Research Unit of General Practice, Department of Public Health, University of Southern Denmark, Odense C, Denmark; c Migrant Health Clinic, Odense Universitetshospital, Odense C, Denmark; d Center for Global Health, University of Southern Denmark, Odense C, Denmark; e The Dartmouth Institute, Geisel School of Medicine at Dartmouth, Hanover, NH, USA; f Direction générale de la santé, Unisanté, Lausanne, Switzerland; g Department of Clinical Research, University of Southern Denmark, Odense C, Denmark

**Keywords:** Co-production, immigrant patient, refugee patient, healthcare service, culturally competent care, patient-provider relationship, person-centred care

## Abstract

**Purpose:**

Immigrant patients run a risk of receiving lower quality of care. Co-production, as the concept of how to collaboratively create valuable healthcare service for the patient, offers a new perspective that might help. The scoping review aimed at identifying and analysing factors facilitating co-production between immigrant patients and healthcare professionals.

**Methods:**

We searched seven scientific databases for peer-reviewed publications of all study designs. Two reviewers independently screened the publications for eligibility and performed data extraction. Data were analysed by applying an inductive, interpretive approach for data synthesis.

**Results:**

Fifteen publications were included for analysis. We identified six factors hat facilitate co-production: 1) prioritizing co-production in the organization, 2) providing a safe environment that promotes trust and patience, 3) using a language the patient understands, 4) respecting the patient’s knowledge and priorities, 5) improvising with knowledge and courage, and 6) engaging in self-reflection.

**Conclusions:**

The scoping review illustrated that co-production with immigrant patients can be successful if the system and professionals are interested and prepared. Immigrant patients could be a valuable source of information and powerful co-producers of their own health. The study contributed to a growing body of research on patient-professional co-production in healthcare and might also prove relevant for other disadvantaged patient groups.

## Introduction

Immigrant patients face major obstacles when accessing the healthcare system in their new homeland (Derose et al., 2007). Limited language proficiency, immigration status, socioeconomic background, stigma, and policies on access to healthcare limit their opportunities to access and be involved in obtaining and using healthcare services (Brämberg et al., 2010). Refugee patients are even more vulnerable due to their experiences with marginalization, poverty, high stress of displacement (Langlois et al., 2016) as well as complex medical problems and trauma history (Adams et al., 2004). As a result, immigrant and refugee patients are at significant risk of receiving lower quality of care (Derose et al., 2007). Healthcare professionals may have insufficient knowledge of cultural issues. Immigrant and refugee patients on the other side are members of a heterogeneous population with a diverse mixture of cultures (Dias et al., 2012). Hence, it may be challenging for healthcare professionals to decode, and invite behaviours that include prejudice, stereotyping, and/or avoidance (Ahmed et al., 2017; Derose et al., 2007). Moreover, these aspects may influence professional behaviour and communication to become more directive, which effectively limits shared decision-making.

Based on the notion that a person’s health is not easily “outsourced” to someone else and that involving patients in their healthcare process can improve the quality of care, healthcare systems are increasingly seeking innovative strategies to create healthcare service in patient-professional partnerships (Coulter & Ellins, 2006). These approaches invite active participation in the medical consultation by asking questions, sharing their resources and social support, and by expressing their concerns and expectations. Immigrant patients, however, are reluctant to engage with the healthcare system and tend to be hesitant when speaking with healthcare professionals (Ahmed et al., 2017). The commonly applied approaches for person-centred healthcare are especially challenging for immigrant patients. The complexity makes it challenging to offer high-quality person-centred healthcare. We need perspectives that will help explore the patient-professional relationship and the co-creation of healthcare services that will offer the best possible value in the healthcare service delivery.

### Co-production of healthcare service

The contemporary dialogue on person-centred care implies that healthcare service is a product, manufactured by healthcare professionals and their systems for patients as consumers. This product-dominant thinking limits the sharing of the professional/patient interdependent work of effectively creating and using a healthcare service. Further, it limits user involvement and constrains the interdependent roles of professionals and users (Batalden et al., 2015). From a service management perspective, all public service is inevitably co-produced in a holistic and dynamic service system, and value is created in a process that is not voluntary but rather intrinsic to the nature of public service itself (Osborne et al., 2016). This view is shared by Batalden et al. (2015) who claim that “*services, unlike products, always are co-produced by service professionals and service users*”. Thus, healthcare service is co-produced by healthcare professionals and patients as a contribution to patient health (Batalden et al., 2015). Co-production recognizes that all people have resources such as their knowledge, skills, habits, and families and communities that can support their effort towards their health and well-being (Loeffler et al., 2013).

**Figure 1.**
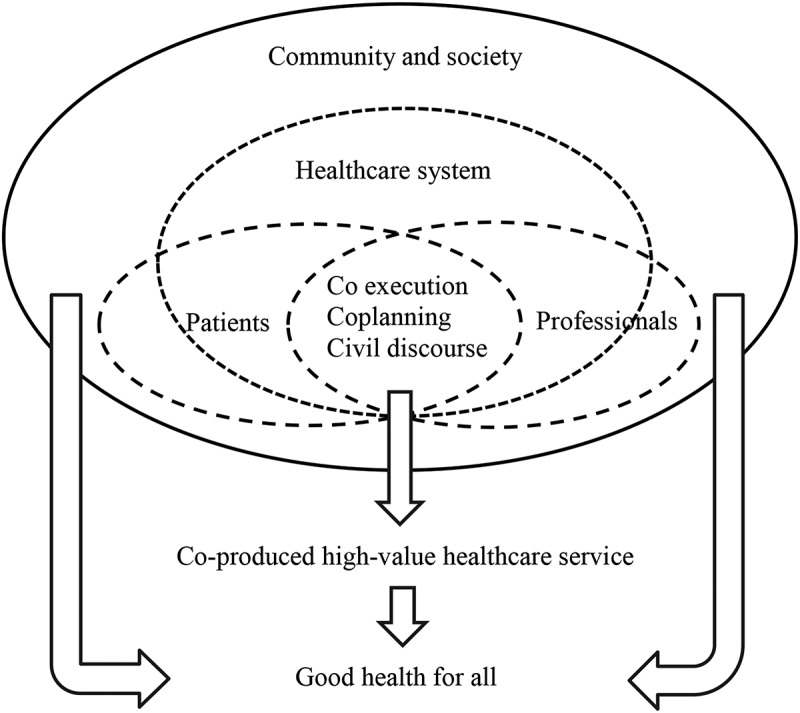
Conceptual model of healthcare service co-production (Batalden et al., 2015)

Batalden’s conceptual model of healthcare service co-production proposes a framework (Figure 1) in which patients and professionals interact as interdependent participants within a healthcare system. At its core, the model recognizes at least three levels of a co-productive relationship. First, (and most basic) good service co-production requires civil discourse with trusting, respectful interaction and effective communication. Second, (and more intensively) shared planning invites a deeper understanding of one another’s expertise, resources, and values. Third, (and most intensively) shared execution demands trust, shared goals, mutual responsibility, and accountability for performance. Moreover, these interactions and relationships are influenced by the structures of the healthcare system as well as by other social forces or services at work in the wider community (Batalden et al., 2015).

Co-production is not a new delivery mechanism for care services. It is an approach that affirms the differences between a “manufactured product” and a “co-created service”. It supports, values, and builds on a collaborative relationship between those persons acting in the roles of the user (patient) and professional (Needham & Carr, 2009). It offers new perspectives on opportunities for healthcare service improvement. It invites a better understanding of the relational and contextual facilitators and support for the work and use of resources (Hardyman et al., 2015). It further invites more investigation of the processes of co-production in practice, including the practices for involving patients and their efforts to improve their own health. Disadvantaged service users (such as immigrant patients) tend to co-produce less due to lack of knowledge and other resources, but if co-production activities are designed to lift these underlying constraints, they may increase both efficiency and equity in the service delivery (Jakobsen & Andersen, 2013). Thus, this scoping review aimed at exploring the existing literature on healthcare service co-production with immigrant patients. The literature search was guided by objectives to identify and analyse factors facilitating the co-production of healthcare service between immigrant patients and healthcare professionals.

## Materials and methods

The scoping review methodology is useful for systematically examining a broad range of evidence to underpin a certain research area and identify implications for policy and practice (Tricco et al., 2016), and was therefore particularly suitable for the purpose of this review study. We previously published a review protocol (Anonymous, 2018) that included more extensive descriptions of the research’s background. This scoping review was reported using the Preferred Reporting Items for Systematic reviews and Meta-Analyses extension for Scoping Reviews (PRISMA-ScR) (Tricco et al., 2018).

### Eligibility criteria

#### Participants

We included both sides of the co-productive relationship, immigrant patients and healthcare professionals, as participants. We searched for literature on immigrant patients of any origin, age, or sex. Refugee patients share many similarities with immigrants in terms of challenges and needs when being in a new country (Bernard, 1976), and were therefore included as well. An ethnic minority is “a group within a country or community which has different national or cultural traditions from the larger, dominant population group” (Oxford English Dictionary, 2020). This includes immigrants, their descendants, and groups of people who were born in a certain country and still belong to an ethnic minority (such as Hispanic and Latino Americans, Native Americans, and Aborigines). Therefore, search terms such as “ethnic minority” or “descendant” were included in the search to include potential useful insights relevant for immigrants. Studies with all types of healthcare professionals were eligible for inclusion.

#### Concept

The core concept examined by the scoping review is the co-production of healthcare service. More specifically, we searched for literature that provided insight into the patient-professional interaction in which they *work together to co-produce* the patient’s health. Co-production as a conceptual name for shared work of healthcare services was only recently introduced to healthcare (Batalden et al., 2015). Therefore, we also included studies on earlier and related conceptual names such as patient-centeredness, patient-professional communication, relationship, involvement, and self-management. We specifically searched for studies that either aimed at establishing a collaborative/co-productive patient-professional relationship or used co-productive principles to reach a certain patient-related goal. They included publications on lessons learned, intervention evaluations, narratives from case studies, or patient/professional experiences. Publications using the concept of participation only for one-sided needs assessment purposes were excluded unless the particular inquiry was on the topic of co-production or the collaborative patient-professional relationship. Further, we excluded publications that mention a need for co-productive/collaborative patient-professional relationships merely as a recommendation, without further details on how to achieve that goal. Because of our focus on the relationship between patient and healthcare professional, we excluded publications concerning patient participation in research.

#### Context

The primary and secondary healthcare sectors served as potential settings for co-production. Primary healthcare settings included for example, be general practitioners, specialists, home care, or nursing homes. Settings in the secondary healthcare sector could be any public, private, somatic, or psychiatric hospital. All types of healthcare services available for patients in ambulatory care, daycare, long-term care were included.

### Information sources and search strategy

The first author designed and conducted the literature search with the support from the author group and two research librarians. An initial search in the PubMed and Scopus databases resulted in 1018 hits in PubMed and 159 in Scopus. We then applied the final keywords and index terms in the PubMed, Scopus, Ovid EMBASE, EBSCO CINAHL, EBSCO PsycINFO, Cochrane Library, and Web of Science databases. We included peer-reviewed journal papers of all study designs, without geographic limits, if they were written in English, German or Scandinavian languages. We identified studies between January 2007 and December 2019. The search year 2007 was selected because around that time “patient-centred care” started to appear frequently in the medical literature as a conceptual name (Laine & Davidoff, 1996). We conducted a follow-up search in July 2020 to check for new studies being published since the first search. The search strategy used in PubMed is illustrated in Appendix_A. Six authors were contacted for further details on their research (three replies received). We used EndNote to remove duplicates and store bibliographic information. All reference lists of the included articles for full-text reading were also screened for additional results.

### Selection of sources of evidence

Citations were transferred to and screened in the web-based screening software Covidence (www.covidence.org). Two reviewers (CR-K, AN) screened titles and abstracts, using the above-formulated eligibility criteria and marked them “include”, “exclude”, or “relevant for other purposes”. This screening process was pilot-tested on twenty articles before the reviewers continued to screen independently. Disagreements were resolved to consensus through discussion and passed to a third reviewer (CvP) for final resolution if the issue could not be resolved. The same two reviewers (CR-K, AN) conducted the full-text screening, which again was pilot-tested on a random sample of five articles.

### Data charting process

Study characteristics extracted were: authors, year, country, aim, patient group, healthcare professional, setting, and the identified co-production element. For results, we abstracted data on factors that may influence the individual co-productive patient-professional relationship or factors related to the healthcare organization and wider community. We searched for co-production as an underlying phenomenon in the interaction and communication between immigrant patients and healthcare professionals. Co-production was not necessarily named per se or an immediate objective of the included studies.

Full data extraction began after a pilot test on a random sample of five articles. Two reviewers each independently abstracted the articles’ characteristics (CR-K, BRT) and results (CR-K, AN), followed by consensus discussions. Discrepancies in findings were resolved in an open and critical dialog. We did not critically appraise the methodological quality of the individual articles, which is consistent with the procedure in scoping reviews (Tricco et al., 2018). Instead, we aimed at prioritizing papers that appeared to be relevant for the review rather than particular study types or methodological standards.

### Synthesis of results

We applied an inductive and interpretive approach to identify factors influencing co-production. Text material was read repeatedly and relevant notes were transcribed inductively. The notes were sorted by identifying recurring themes and then grouped into categories and subcategories. In this process, coding evolved from being simply descriptive to be more interpretive. Data were analysed and categorized by one author (CR-K) as the team engaged in an ongoing discussion of results. The final list of factors influencing co-production was consolidated in team discussions. After discussing the results, the identified factors from the inductive analysis were linked to the core concepts of Batalden et al’s co-production model (Batalden et al., 2015).

## Results

### Search results and included studies

Based on our search strategy, we retrieved a total of 3394 peer-reviewed studies. After removing duplicates and screening titles and abstracts, 50 full-text articles were retrieved. Of these, 35 articles did not meet the inclusion criteria. Most of them (n = 26) were excluded because they did not contain details on co-production as the *joint co-production activity* between patient and professional. We included 14 peer-reviewed articles (Balachandra et al., 2009; Balán et al., 2013; Benson et al., 2010; Borkan et al., 2008; Brassart et al., 2017; Cochran et al., 2017; DeCamp et al., 2015; Kaltman et al., 2016; King et al., 2015; Lee et al., 2009; Mead et al., 2013; Mendenhall et al., 2008; Naldemirci et al., 2018; Polo et al., 2012) for further analysis.

**Figure 2.**
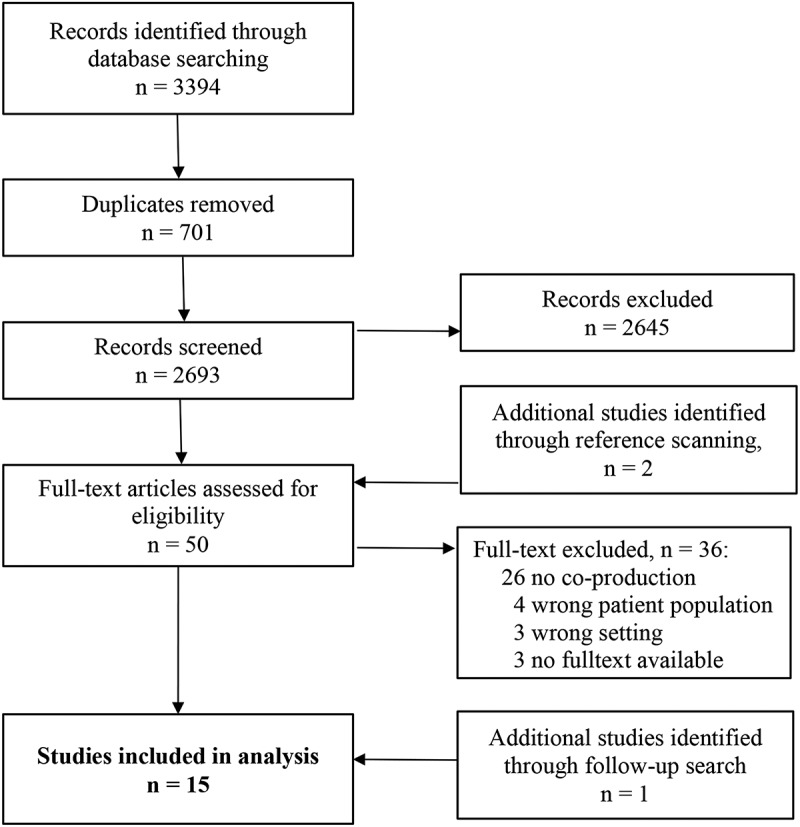
PRISMA flowchart showing the study selection process

A follow-up search before submitting the manuscript resulted in one additional article to be included (Alegria et al., 2020). The study selection process is presented in the PRISMA-ScR flowchart in Figure 2.

Of the 15 included scientific articles, nine were from the USA. The remaining five articles were from Australia, Canada, and Sweden. The majority (n = 12) of studies were based on primary research. Patient groups were described as being migrant/immigrant patients (n = 6), ethnic minority/Latino patients (n = 6), and refugee patients (n = 3). All articles involved nurses and/or physicians as healthcare professionals. Sometimes third parties were mentioned such as outreach coordinator, interpreter, social workers, and bilingual care manager. Six articles took place in a hospital setting, six in a primary healthcare setting, and three in a mental healthcare setting. More than half of the articles (n = 8) described the implementation of new (co-productive) approaches to e.g., improving healthcare services, self-management, or patient involvement. The remaining articles described experiences with established routines built on co-productive principles. Study characteristics are presented in Table I.

**Table I.** Characteristics of included studies

Authors Year	Country	Aim of the article	Patient group	Healthcare professionals	Setting	Co-production element
**Intervention studies**						
(Balán et al., 2013)	USA	Describe experiences with a motivational pharmacotherapy intervention	Spanish-speaking immigrant patients	Psychiatrists	Psychiatric clinic	Co-productive counselling to improve treatment adherence
(DeCamp et al., 2015)	USA	Describe experiences with family advisory groups for clinics and hospitals	Latina mothers	Healthcare professionals	Primary healthcare setting	Identify and address areas for healthcare improvement in a co-productive process
(Lee et al., 2009)	Australia	Describing experiences of a consumer reference group to understand barriers for migrant patients	Ethnic women	Healthcare professionals	Primary healthcare setting	Patient participation in primary health care programme planning and services
(Kaltman et al., 2016)	USA	Measuring the effectiveness of an integrated self-management intervention	Latino patients	Healthcare professionals	Primary healthcare setting	Co-production of an intervention for diabetes and depression self-management
(Polo et al., 2012)	USA	Describing benefits and challenges of strategies aiming at increasing patient participation	Latino patients	Bilingual care managers, healthcare professionals	Mental healthcare setting	Individuals’ contribution to co-production of healthcare service
**Interview studies**						
(King et al., 2015)	Canada	Examining strategies to enhance culturally sensitive care in paediatric rehabilitation	Immigrant parents of children with disabilities	Social workers, occupational and speech therapist	Hospital setting	Tools for and approaches to co-productive culturally sensitive care
(Brassart et al., 2017)	Canada	Describing barriers to and strategies for treatment engagement	Immigrant parents raising a child with a disability	Healthcare professional	Hospital setting	Engagement in and understanding of the therapeutic process of immigrant parents raising a child with a disability
(Naldemirci et al., 2018)	Sweden	Describing the risks of person-centred care in three research projects	Migrant patients	Healthcare professionals	Healthcare setting (used for community setting)	Co-production in person-centred care routines (patient narrative, partnership, and documentation)
**Case studies**						
(Mendenhall et al., 2008)	USA	Describing the clinical journey of a refugee patient	Refugee patient	Healthcare professionals, outreach coordinator	Primary healthcare setting	A co-productive partnership between patient and healthcare professionals
(Balachandra et al., 2009)	USA	Describing the clinical journey of a refugee patient	Refugee patient	Healthcare professionals, Interpreters	Primary healthcare setting	Co-production between patient and health care professionals
(Cochran et al., 2017)	USA	Describing the tragic medical case of a Pakistani immigrant family	Immigrant parents to a terminally ill child	Healthcare professionals	Hospital setting	Cultural humility in shared decision-making
**Systematic review**						
(Mead et al., 2013)	USA	Review of literature on shared-decision making	Minority patients	Healthcare professionals	Hospital setting	Factors influencing co-production with minority patients
**Other publications**						
(Borkan et al., 2008)	USA	Perspective paper describing a framework for quality care that relates to ethnicity-based on case examples	Migrant patients	Healthcare professionals	Hospital setting	Approaches and tools to achieve co-productive cultural competency and humility
(Benson et al., 2010)	Australia	Editorial describing a refugee’s experiences of mental health services	Refugee patient	Healthcare professionals	Hospital setting	The role of narrative competence in co-producing a patient’s diagnosis and treatment
(Alegria et al., 2020)	USA	Editorial describing lessons learnt about how to engage ethnic minorities	Minority patients	Healthcare professionals	Mental healthcare setting	The role of egalitarian collaboration in the clinical encounter

### Six factors in the co-production of healthcare services with immigrant patients

We identified six factors in the work that facilitate co-production with immigrant patients (Table II). In the usual temporal order of a healthcare service, factors one and two should be done before, step three, four and five during, and step six during and/or after the direct interaction between an immigrant patient and the healthcare professional.

**Table II.** Factors towards co-production with immigrant patients

Nr.	Factor	Underlying strategy	How to exactly?
1	**Prioritizing co-production in the organization**	Create consensus on co-production across all organization levels Allow creativity amongst people working on the frontline	No “tokenistic gesture” Allocating time and other resources Having organizational practices and habits for co-production
2	**Providing a safe environment that promotes trust and patience**	Signalizing “I see you, I hear you and I try to understand you”	Allowing trust to be built little by little Exercising patience
3	**Using a language the patient understands**	“Tune in” on how to communicate with the patient	Taking time for friendly, simplified communication Using a medical competent interpreter
4	**Respecting the patient’s knowledge and priorities**	Create a shared starting point by listening to the patient’s story and worries	Learning about the underlying cultural background for patient’s priorities Considering family situation Acknowledging the patient’s resources and other priorities Including family/society context
5	**Improvising with knowledge and courage**	Be creative in meeting the patient’s needs	Tailoring interventions to the patient’s reality Including approaches outside the biomedical paradigm Accepting the open-ended process
6	**Engaging in self-reflection**	Take a step back and do not let harmful stereotypes affect your judgement	Being aware of stereotypic views, own norms, and values Asking questions Learning about other cultures

#### Prioritizing co-production in the organization

From the included studies it became apparent that a key facilitator for co-production was the healthcare organization’s willingness to be flexible and actively support an approach that recognizes the diversity of patients in terms of background, prior experiences, and nature of the problem (Balachandra et al., 2009; Brassart et al., 2017; Cochran et al., 2017; King et al., 2015; Lee et al., 2009; Mendenhall et al., 2008). First and foremost, this required a shared commitment to the “*why*” and “*how-to*” to co-produce from both management (top-down) and healthcare professionals (bottom-up). This commitment also implied, having an agreement that allowed co-production to become an integrated part of the organizational structures instead of treating it as a “tokenistic gesture” operating outside the system (Lee et al., 2009; Mendenhall et al., 2008).

Organizational support was accompanied by resources and time allocated for a range of new (and maybe unusual) organizational practices and habits: allowing longer/more frequent consultations (Balachandra et al., 2009; Brassart et al., 2017; King et al., 2015; Lee et al., 2009; Mead et al., 2013); flexible meeting times (Lee et al., 2009; Mendenhall et al., 2008); having the possibility of using interpretation services (Balachandra et al., 2009; Borkan et al., 2008; Brassart et al., 2017; DeCamp et al., 2015); guaranteeing continuity in care (Balachandra et al., 2009; Lee et al., 2009); and in some cases even doing home visits (Brassart et al., 2017). Displaying flexibility when engaging with a patient in new ways (King et al., 2015; Lee et al., 2009) or coordinating collaborations with other disciplines (Balachandra et al., 2009; Lee et al., 2009; Mendenhall et al., 2008) also strongly facilitated the co-production of healthcare service.

#### Providing a safe environment that promotes trust and patience

Before the patient and the professional could reach a state at which it was natural for them to find solutions together, they needed to create a safe environment for sharing one’s viewpoints and concerns (Alegria et al., 2020; Balachandra et al., 2009; Benson et al., 2010; Brassart et al., 2017; Cochran et al., 2017; DeCamp et al., 2015; Lee et al., 2009; Naldemirci et al., 2018; Polo et al., 2012). In this process of getting to know one another, patients became more familiar with the dynamics of the healthcare system. The professionals on the other side had the unique opportunity to become culturally aware and learn more about a patient and his/her background story (Alegria et al., 2020; Benson et al., 2010; Brassart et al., 2017). In several studies, being prepared to exercise patience and allowing trust to build incrementally seemed to result in more engaged and active patients (Balachandra et al., 2009; Benson et al., 2010; DeCamp et al., 2015; Lee et al., 2009; Polo et al., 2012). With time, they started to feel more comfortable to give honest feedback, talk more openly, and ask questions (Balachandra et al., 2009; DeCamp et al., 2015). Carrying out simple acts of kindness, empathy, respect, and even humour showed to be essential tools when working across cultures and also include the professional as part of the therapy (Benson et al., 2010; Cochran et al., 2017).

Ultimately, it was the message of “*I see you, I hear you and I try to understand you*” that contributed to giving the patient the feeling of being respected and listened to. To create a safe environment for the patient to tell his story was hereby an important starting point for any collaborative relationship.

#### Using a language the patient understands

Communicating in a language the patient understands was described in more than half of the included articles as a key prerequisite for successful co-production patient and professional (Alegria et al., 2020; Balachandra et al., 2009; Benson et al., 2010; Borkan et al., 2008; Brassart et al., 2017; King et al., 2015; Lee et al., 2009; Mead et al., 2013; Polo et al., 2012). The importance of communication began when welcoming the patient. Simple acts of kindness, such as asking a few personal questions at the beginning of the conversation, could make a big difference in starting the conversation (Benson et al., 2010; Brassart et al., 2017; King et al., 2015). Professionals in the included publications would calculate extra time to explain, re-explain if necessary, ask questions to ascertain understanding, or give time to absorb information and room for the patient to ask questions (Balachandra et al., 2009; Brassart et al., 2017; King et al., 2015; Polo et al., 2012).

We identified a range of communication strategies used in the included articles: a) speaking slowly, clearly and using simple words without jargon (Alegria et al., 2020; Brassart et al., 2017; King et al., 2015; Lee et al., 2009); b) using simple tools such as written down instructions, visual materials as well as using gestures and metaphors (Brassart et al., 2017; King et al., 2015); and c) paying attention to non-verbal communication by observing behaviour and reactions that could indicate if more explaining or time for absorption is needed (Borkan et al., 2008; Brassart et al., 2017).

Medically competent interpreters were often valuable assets in the co-production process, especially if they supported decoding mutual cultural differences instead of simply doing verbatim language translation (Balachandra et al., 2009; Borkan et al., 2008; Brassart et al., 2017; Mendenhall et al., 2008). Having the opportunity to speak in one’s mother tongue increased mutual understanding and gave the patient a sense of security that contributed to strengthening the patient-professional relationship (DeCamp et al., 2015; Mendenhall et al., 2008). Despite being able to speak another language, feelings and beliefs were best expressed in one’s first language (Benson et al., 2010). Thus, both sides obtained more knowledge about each other’s culture (e.g., patient’s cultural concerns or organizational routines in the healthcare system), and with which they might not have been familiar (Brassart et al., 2017; Mendenhall et al., 2008).

Basically, healthcare professionals in the included studies seemed to make an effort on “tuning in” on how the patient felt on the day, existing language and communication skills, how much explanation work was needed, and how much information the patient could absorb. They seemed to be getting a feeling of how to talk to one another before even starting the therapeutic process.

#### Respecting the patient’s knowledge and priorities

In several studies, we observed the importance of being curious about the patient’s story. Professionals practiced wilful exploratory listening to try to learn not only about the patient’s priorities but also the underlying cultural background for these priorities (Alegria et al., 2020; Balán et al., 2013; Benson et al., 2010; Borkan et al., 2008; Cochran et al., 2017; King et al., 2015; Mendenhall et al., 2008; Naldemirci et al., 2018; Polo et al., 2012). Not all patients desired a participatory approach (Brassart et al., 2017; Lee et al., 2009), but co-production happened, where patient and professional jointly and collaboratively determined their respective roles (Brassart et al., 2017; King et al., 2015; Polo et al., 2012) and co-created an understanding of the patient’s situation as well as how to move forward (Borkan et al., 2008; Brassart et al., 2017; Kaltman et al., 2016; King et al., 2015; Naldemirci et al., 2018).

As the included studies showed, successful co-productive relationships were informed by efforts to redress or monitor the imbalance of power between patient and professional by sharing power, knowledge, expertise, and joint goal setting (Alegria et al., 2020; Balán et al., 2013; Borkan et al., 2008; Brassart et al., 2017; Kaltman et al., 2016; King et al., 2015; Naldemirci et al., 2018; Polo et al., 2012). This process was further reinforced by helping the patients see their viewpoints and contributions being worthwhile (Balachandra et al., 2009; Benson et al., 2010; Kaltman et al., 2016; Lee et al., 2009; Mead et al., 2013; Mendenhall et al., 2008) or by acknowledging alternative resources such as religious faith as a deep source of strength (Benson et al., 2010; DeCamp et al., 2015; Mead et al., 2013) or the social context such as family and the wider community (Balachandra et al., 2009; Benson et al., 2010; Brassart et al., 2017; Cochran et al., 2017; King et al., 2015; Mead et al., 2013; Naldemirci et al., 2018). For some patients, decision-making and the therapeutic process were a collective experience shared with their families (“family-centred decision-making”). The cultural norm of collectivism played a positive role through the support and involvement of the family. It could, however, also play an adverse role if increased family involvement leads to treatment delay and limited disclosure of the condition to the family (Mead et al., 2013).

In numerous studies we noticed the importance of acknowledging other priorities, immigrants might be having in their life and that left them practically incapable of taking action (Benson et al., 2010; Brassart et al., 2017; King et al., 2015). This included struggles related to getting acculturated to a new country, culture, language or even food, settlement issues related to housing, living and employment, isolation, grief, and guilt for leaving one’s family, and poverty. Reaching a shared understanding of what to do, the healthcare professionals had to be aware of their own cultural background and how the patients’ underlying cultural background was related to their opinions and preferences.

At times, healthcare professionals might find their norms and values challenged when facing ethical dilemmas; for example, when being asked by a patient (or patient caregiver) to withhold important information or bad news from the family and others, to either shield them from pain (Cochran et al., 2017) or due to stigma related to their health problem and the associated fear of being discriminated (Benson et al., 2010; Mead et al., 2013).

#### Improvising with knowledge and courage

In several publications, tailored interventions took the patient’s reality into account by respecting cultural and individual preferences (Balán et al., 2013; Kaltman et al., 2016; King et al., 2015; Mead et al., 2013; Mendenhall et al., 2008). Sometimes, usual values and routines of modern biomedicine (Benson et al., 2010; Borkan et al., 2008) or traditional models of shared decision-making (Mead et al., 2013), and creative or improvised approaches were chosen as the way forward. These tailored interventions looked at cultural barriers as opportunities for enhanced personalized treatment (Cochran et al., 2017), and might be more (cost)effective than passively receiving professional services (Mendenhall et al., 2008). Examples for unconventional alternative approaches were: intentionally replacing typical routines with interaction to improve communication (Balán et al., 2013; Kaltman et al., 2016), choosing a holistic treatment that is embedded in a patient’s cultural context and not based on diagnostic codes (Mendenhall et al., 2008), or simply including a patient’s family in the decision-making process (Mead et al., 2013).

Being both knowledgeable and creative, improvisation meant trusting an open-ended process that at times could be “rich and messy”. It involved for instance, leaving room for change for patients to determine the direction forward (Balachandra et al., 2009; Balán et al., 2013; Lee et al., 2009; Mendenhall et al., 2008). This implied pacing with patients, not pushing, but guiding them with one’s professional opinion so that patients did not find themselves in a defensive position (Balán et al., 2013; King et al., 2015).

#### Engaging in self-reflection

In numerous studies, healthcare professionals took a step back to not let harmful stereotypical thinking about patients’ beliefs, norms, and backgrounds, affect their judgement (Benson et al., 2010; Borkan et al., 2008; Cochran et al., 2017; Naldemirci et al., 2018; Polo et al., 2012). On another note, a silent patient was not necessarily a patient that does not want to communicate. Silence could also be a sign for patients to be feeling intimidated by the fact that they could not interact as they were used to (Benson et al., 2010; King et al., 2015). Here, healthcare professionals would apply exploratory listening and active questioning to overcome erroneous assumptions that arose from areas left unexplored (King et al., 2015). Others engaged in learning about different illness explanatory models since somatization in many cultures is the only way of expressing psychological distress (Benson et al., 2010); others tried to find the middle ground between treating each patient as a unique individual and seeing them as a person embedded in a “typical” view of their social and cultural context (Naldemirci et al., 2018).

## Discussion

Analysing the 15 included studies, we created an overview of factors that seemed to facilitate a co-productive relationship between immigrant patients and healthcare professionals in primary, hospital, and mental healthcare settings. The studies provided insight into how a co-productive relationship could be fostered through preparations, practices, and routines before, during, or after the direct patient-professional interaction. We identified six facilitating factors of work towards healthcare co-production with immigrant patients: 1) prioritizing co-production in the organization; 2) providing a safe environment that promotes trust and patience; 3) using a language the patient understands; 4) respecting the patient’s knowledge and priorities; 5) improvising with knowledge and courage; 6) engaging in self-reflection.

Co-producing healthcare service challenges “total” standardization by letting patient and family priorities dominate the delivery of healthcare service (Batalden et al., 2015). Therefore, ***prioritizing co-production in the organization*** (factor 1) across and between management and healthcare professionals is one of the foundation stones for intentional co-production. Of the included literature, only a few studies described experiences with established and integrated routines influenced by co-productive principles. Co-production is a description of the shared work of making a service between patient and healthcare professional. Therefore, co-production is done in many ways at the operational level in both primary healthcare and hospital settings. It is not an add-on to the delivery of public service but a core element of effective management of public service on a day-to-day operational basis (Osborne & Strokosch, 2013) that requires sufficient resources, such as a) organizational flexibility with sufficient time and resources, b) good interpreting services, and c) collaborating with social services and families if needed (Priebe et al., 2011). Despite some scepticism about the resource effectiveness of co-production (Holland-Hart et al., 2019), allowing healthcare services to become shaped and tailored by co-productive principles might help avoid costly mistakes and be a time- and cost-efficient way of meeting a patient’s needs (Elwyn et al., 2019).

In healthcare co-production, the agreement of the professional’s role is key. The professional can support, encourage, and coordinate the co-production capability of the users of a service (patients) and the communities in which they live (Bovaird, 2007). This facilitates moving beyond tokenism and towards shared power and decision-making. Thus, staff needs to be trained and supported within an organizational context where the value of collaborating with a broad diversity of patients is clear, embedded, and normal instead of simply involving a narrow and “easy to work with” group of individuals (Ocloo & Matthews, 2016). This is because “totally” standardized solutions as *manufactured goods provided to* a patient are still very much alive and engrained in our managerial mindsets (Joiner & Lusch, 2016).. Ultimately, prioritizing people and relationships rather than tight organizational procedures leads to a compassionate organization which according to Greenhalgh (2013) “supports and shapes compassionate behaviour by its members, partly through appropriate incentives, rewards, and procedures but mainly by recognizing that emotions—feeling, caring, loving, yearning—are an integral component of our rationality, not something that distorts or detracts from it”. A compassionate organization recognizes that “kindness is a binding, creative, and problem-solving force that inspires and focuses the imagination and goodwill. It inspires and directs the attention and efforts of people and organizations towards building relationships with patients, recognizing their needs, and treating them well. Kindness is not a ‘nice’ side issue in the project of competitive progress. It is the ‘glue’ of cooperation required for such progress to be of most benefit to most people (Ballatt et al., 2020)”.

A ***safe environment that promotes patience and trust*** (factor 2) is another prerequisite for co-producing healthcare service with immigrant patients. Trust is not a static attribute but develops reciprocally as a result of building relationships with one another supported by strategies such as emotional/physical closeness, humour, or mutual respect (Wilson et al., 1998). Many patients value a trusting relationship and positive attention more than professional competences and expertise. To build trust, patients rely on simple and honest signals, and practices such as deep inquiry, careful listening, and the display of emotions and fears (Riva et al., 2014). Positive and trustful encounters are particularly important for patients with different cultural backgrounds as they might *a priori* trust health professionals to a lesser extent (Doescher et al., 2000), based on prior traumatic experiences in their country of origin or during the flight (Benson et al., 2010). Others have reservations towards the healthcare system because of previous negative healthcare service experiences (Naldemirci et al., 2018). Some immigrant patients, on the other hand, feel compelled to trust the professional because cultural and language barriers discourage their questioning of recommendations or active involvement in their treatment (Hillen et al., 2018). Due to the reciprocity of trust, healthcare professionals must be prepared to trust the decisions and behaviours of service users rather than attempt to dictate them (Bovaird, 2007) even though this implies vulnerability and interdependency on the side of healthcare professionals (Hupcey et al., 2001). Mastering this challenge, can enhance relationships and collaboration and have a positive impact on professional well-being and satisfaction (Pellegrini, 2017).

Cultural and linguistic differences between professionals, patients, and their families add further complexity to the quest of ***finding a language the patient understands*** (factor 3) during the clinical encounter (O’Toole et al., 2019). Healthcare professionals often cannot rely on habitual ways of communicating with patients. They have to “tune in” to the immigrant patient partner by mobilizing listening skills, empathy, and attention to the components of communication that are frequently neglected such as para-verbal and non-verbal clues (Ranjan et al., 2015). This is especially relevant for interacting with people from certain cultural backgrounds (most countries in Asia, Africa, the Middle East, and South America) who are looking for exactly these clues—body language, gestures, facial expressions or changes in voice—when filtering meaningful information out of a conversation (Hall, 1981).

Doctors especially have been trained to maintain a professional distance to avoid emotional involvement that might compromise science-informed observation and practice (Hardy, 2017). However, expressing empathy clearly is beneficial since it allows patients to talk more about their symptoms and concerns, thus enabling the physician to collect more detailed medical and psychosocial information (Coulehan et al., 2001). It also contributes to a greater sense of fulfilment and reduces the risk of professional burnout especially among physicians (Hardy, 2017).

Medical interpreters play an important role in communication and co-producing with immigrant patients. Skilled medical interpreters are cultural brokers that use their cultural competences to include the wider social context of the person in their translation of the conversation (Gustafsson et al., 2013). Hereby, they facilitate mutual understanding and strengthen the relationship between patient and healthcare professionals as well as contribute to shaping the agenda of the encounter (Baraldi & Gavioli, 2013; Bischoff et al., 2012). However, healthcare professionals need training and preparation to communicate effectively through interpreters (Clarke et al., 2019). They need to consider the set-up of the consultation room and their ways of speaking. As a pragmatic facilitating step, they might check in with the patient and interpreter from time to time to make sure that the encounter is going well or if any issues should be addressed (Clarke et al., 2019).

Co-production has to take into account ***an immigrant patient’s knowledge and priorities*** (step 4). For healthcare professionals, it is unrealistic to be knowledgeable about all immigrant patients’ different cultural and ethnic backgrounds and social supports. So, the professional’s attitude towards the immigrant patient and the willingness to accept an immigrant’s cultural background is key (Whittal et al., 2017). This includes insight into the patient’s willingness and ability to adapt to and finally integrate into a new culture. Important factors to consider are the resettlement process and the learning and supports “surviving” in new surroundings (Gonsalves, 1992), the personality, experiences before/during/after migration, and prior traumatic experiences (Perreira & Ornelas, 2013).

A good relationship and communication between professionals and immigrant patients and their families may have its foundation in their “cultural” attitudes (combined acculturation orientations) towards one another, which may contribute to the quality of care, health behaviours and ultimately the quality of life of the immigrant (Whittal et al., 2017). The complexity of the cultural and biographical background of immigrant and refugee patients are strong advocates for explicit efforts in the co-production of healthcare. The need to access and incorporate sources of information that might be unknown or even alien to the healthcare professional puts the patient in a powerful and knowledgable position. Including the unique and contextual way of how a patient experiences illness does not require the professional to reject the principles of evidence-based medicine. It rather presupposes an interpretive approach from the healthcare professional’s side to meaningfully draw on all aspects of evidence (Greenhalgh, 1999). Unfortunately, healthcare professionals are already all too often under pressure to “deliver” as many cures as possible. Confronted with a patient’s narrative, they often find themselves at an intersection between objective classifications and subjective stories. This dilemma should be approached by sharing and negotiating both types of knowledge—professional and patient—in the quest for co-produced solutions (Launer, 1999).

Healthcare professionals often need ***to improvise with knowledge and courage*** (factor 5) to co-produce health care that meets the needs of an immigrant patient. Guidelines for science-informed practice may be difficult to apply. Medical consultations are rarely simple, and uncertainty about the best solution is common. Problems are undifferentiated and symptoms vague (Innes et al., 2005). This applies even more to heterogeneous groups of immigrant patients with language as well as cultural and psychological barriers. They might have different needs for participating in their care. For some, participation means to be listened to and taken seriously; others participate by choosing from different treatment options (Brämberg et al., 2010). Standardized roles and routines, governed by tight norms and rules need to be balanced with individual customization. Here, co-producing healthcare inevitably is a holistic approach, implying tailoring processes and services to the understanding, beliefs, wishes, and needs of the patient and the family (Greenfield et al., 2018). In other words, healthcare professionals have to make sense of an immigrant patient’s narrative and integrate this knowledge in the usual work processes in healthcare. Weick (1995) describes this process of sensemaking as the “diagnostic process directed at constructing plausible interpretations of ambiguous cues that are sufficient to sustain action”. Embracing uncertainty and unpredictability may enable healthcare professionals to improvise and to find creative solutions with their immigrant patients (Innes et al., 2005).

***Engaging in self-reflection*** (factor 6) is a crucial component in providing and maintaining quality care. Pre-established assumptions or stereotypic views about immigrant patients’ behaviour, norms, or preferences could lead to too much or too little (Borkan et al., 2008; Cochran et al., 2017). Some healthcare professionals try to avoid stereotyping by claiming to be socially and culturally neutral and treating each patient without any assumptions at all. This view makes them effectively deny the role of sociocultural factors that affect both the patient and themselves (Beagan & Kumas-Tan, 2009). Therefore, self-reflection concerning immigrant patients includes being aware of and humble about how a person’s culture can impact health behaviours. This awareness can be used to cultivate sensitive approaches in treating patients (Prasad et al., 2016).

In general, being able to reflect, requires the skill to notice the prompt or trigger that can help stimulate reflection. This prompt—often a dilemma or moment of uncertainty about what should be done—can lead to exploring and challenging one’s underlying assumptions, beliefs, motives, and values (Murdoch-Eaton & Sandars, 2014). Also, it can stimulate critical reflection on how to improve one’s competence and do things differently the next time (Fragkos, 2016).

Informal positive feedback and patient narratives can also be powerful triggers for reflection (Jones et al., 2020). Positive feedback on patient experience such as thank you cards or hugs not only impacts healthcare professionals’ reflections on what they had done well but also valorizes their work. Hearing patients’ stories can also stimulate reflection since it challenges healthcare professionals to think more deeply about their own attitudes and behaviours, and as a result, adjust their approach to communicating and interacting with patients. Although, informal feedback can be seen as highly powerful, in practice it is rarely used systematically and thus, left to the individual to recognize it as a trigger for reflection (Jones et al., 2020).

### Linkage to the conceptual model of healthcare service co-production

The relationship and interaction between patient and professional lies at the core of the conceptual model of healthcare service co-production. The model also explicitly recognizes the important support of both the healthcare system and the community (Batalden et al., 2015). The six identified factors in the co-production with immigrant patients are very well aligned with and support the principles of the conceptual model.

The importance of having explicit values and practices that support co-production (factor one) in the *healthcare system* was explicitly described above. The relevance of *community and society* focuses on understanding the patient, potential other stress factors and distractions, cultural aspects, and the support systems that influence a patient’s ability and willingness to co-produce. *Civil discourse* could be linked to several factors in our results. Providing a safe and trustworthy environment requires a civil discourse based on respectful and trusting patient interaction (factor two). Furthermore, civil discourse with immigrant patients requires effective communication with adjusted communication strategies and tools as well as the use of medically competent interpreters (factor three). Civil discourse also comes into play when creating a shared understanding of the story, worries, and preferences of the patient (factor four). Last, reflecting on one’s assumptions and beliefs can trigger healthcare professionals to improve their competences and thus contribute to a better civil discourse (factor six). *Co-planning* invites a deeper understanding of one another’s expertise, values, and accessible resources. The last core concept of *co-execution* relates directly to improvising with knowledge and courage (factor five). Healthcare professionals often have to choose alternative sometimes unconventional, innovative approaches based on shared goal-setting, resource availability and the work to be done by patient and professional to tailor an intervention to the situation.

### Strengths, limitations and methodological considerations

To our knowledge, this review was the first to systematically retrieve and summarize the literature on healthcare service co-production with immigrant patients. Despite the increasing interest in co-production research around the world (Bovaird et al., 2019), all included publications originated from (statistically) more developed parts of the world. This might be associated with the fact that more than half of all international migrants live in Europe and Northern America. Also, over the last ten years, major migration streams consisted of refugee movements, reflecting crises, conflicts, or instability (United Nations, 2019). Nine of the 14 included articles were from the USA. This might be the result of including ethnic minorities (such as Hispanic and Latino Americans) in the search strategy. These limitations might limit the transferability of the scoping review findings. They however also show the need for more research on individual healthcare service co-production with vulnerable patient groups such as immigrants and refugees in other countries. This scoping review is based on a western research perspective. Here, it would be valuable to conduct similar research on co-production in collaboration with researchers from the immigrants’ countries of origin.

The concept of co-production lacks a single, universally applicable definition. It is used in a variety of collaborative arrangements in different contexts involving a wide range of actors and benefits from an operational definition when used (Osborne & Strokosch, 2013). The term is newly applied in healthcare. For these reasons, it was challenging to build a well-balanced search strategy that captures relevant historical literature. We struggled to create our operational definition of the term and did not want a definition that forced us to overlook relevant publications. On the other hand, we needed a clear frame to find relevant publications.

We also screened the literature for related concepts such as patient-professional communication, collaboration, and participation. These neighbouring concepts often overlapped with co-production but the origins and use of the terminologies are different. In retrospect, we realized that including search terms such as “culturally competent care” and “transcultural care” might have further strengthened the internal validity of our results since co-production with immigrant patients to a great extent is about culturally competent care. The screening and inclusion phase of our study turned out to be an interpretive process. Often, the dynamics behind co-productive processes between patient and healthcare professional were either hidden between the lines or not at all described in sufficient detail in the historical literature sources. Therefore, the presence of two reviewers was essential in order to ensure internal validity and avoid reviewer bias. Some publications could be excluded because their view of participation did not move beyond “consulting” patients on their perspectives without involving them in finding a co-produced solution. Due to the time gap between the first literature search and manuscript submission, we did a second search which resulted in one additional article to be added to the review. The additional study did not present new insights, that were not already raised in the result section before.

We only included peer-reviewed publications in scientific databases. Searching unpublished literature on co-production and narrative accounts of immigrants in healthcare would most probably provide a broader range of results. However, searching this amount of unpublished and non-peer-reviewed literature without geographic limitation goes beyond the scope of this study. Searching unpublished literature in only one or two geographical areas, on the other hand, would have coloured the results due to differences in medical culture and systems.

The lack of quality appraisal of the included publications is a clear limitation of scoping reviews. It is further complicated by the quality criteria used. However, our aim was not to find the best quality evidence but to provide an overview of the evidence available in a still scattered described area of research within healthcare.

## Conclusion

In this scoping review, we set out to identify and analyse factors that facilitate a co-productive relationship between immigrant patients and healthcare professionals. Against common belief that immigrant and refugee patients might be less likely to engage in their own healthcare, we found evidence that immigrant patients and professionals could co-produce healthcare service. They did so by collaboratively building on the patient’s narrative and/or solution for the patient’s situation. We present the results of the review as six factors that might be considered before, during and after the direct interaction between patient and professional: 1) prioritizing co-production in the organization; 2) providing a safe environment that promotes trust and patience; 3) using a language the patient understands; 4) respecting the patient’s knowledge and priorities; 5) improvising with knowledge and courage; 6) engaging in self-reflection. These factors expand known recommendations for relationships between healthcare professionals and patients. To include the heterogeneous group of immigrant patients added useful new insight for co-producing with a patient group that healthcare professionals often find difficult to approach, communicate, collaborate, and find solutions with.

To our knowledge, this was the first study to explore the concept of co-production of healthcare services with immigrant patients. The results contribute to the growing body of research on how patient-professional co-production can be established, implemented, and create value in terms of advancing health problems and contributing to patient wellbeing. Further research should explore the processes, values, and dynamics of co-production between immigrant patients and healthcare professionals in different geographic contexts and health care settings. Ethnographic field research informed by these factors and specifically focusing on the co-production dynamics between immigrant patients and professionals offers one such possibility. Healthcare professionals’ development might explicitly offer attention to the development of useful knowledge, skill, and habits. Organizational leadership might explore the creation of conditions that offer a better understanding of the rich sources of information that immigrant patients offer in their search for ever better value services and fewer costs of wasteful interventions. Finally, when the system and healthcare professionals prepare well, immigrant patients seem able to be powerful co-producers of their own health.

## Appendices

**Appendix A.** PubMed search strategy and results

9 December 2019		Title/abstract
Block 1		
1	Coproduction OR co-production OR coproduce OR co-produce OR coproducing OR co-producing	2.269
2	Cocreation OR co-creation OR cocreate OR co-create OR cocreating OR co-creating	616
3	Codesign OR co-design OR codesigning OR co-designing	678
4	Cooperation OR co-operation OR cooperate OR co-operate OR cooperating OR co-operating	70.116
5	Collaboration OR collaborate OR collaborating	79.684
6	Co-care	18
9	“patient participation” OR “patient involvement” OR “patient empowerment” OR “patient activation”	6.620
10	“Relationship-centered care” OR “relationship-centred care”	174
11	“patient-centered care” OR “patient-centred care” OR “patient-focused care”	6.912
12	“patient-centered nursing” OR “patient-centred nursing”	85
13	“patient-centered communication” OR “patient-centred communication”	639
14	Patient-centeredness OR patient-centredness	1.394
15	“shared decision-making”	7.495
16	“cross-cultural communication” OR “culturally competent care”	803
17	“patient-provider relation” OR “patient-provider relations” OR “patient-provider relationship” OR “patient-provider relationships” OR “patient-provider communication” OR “patient-provider communications” OR “patient-provider interaction” OR “patient-provider interactions”	2.243
18	“patient-physician relation” OR “patient-physician relations” OR “patient-physician relationship” OR “patient-physician relationships” OR “patient-physician communication” OR “patient-physician communications” OR “patient-physician interaction” OR “patient-physician interactions”	2.392
19	“patient-doctor relation” OR “patient-doctor relations” OR “patient-doctor relationship” OR “patient-doctor relationships” OR “patient-doctor communication” OR “patient-doctor communications” OR “patient-doctor interaction” OR “patient-doctor interactions”	883
20	“patient-nurse relation” OR “patient-nurse relations” OR “patient-nurse relationship” OR “patient-nurse relationships” OR “patient-nurse communication” OR “patient-nurse communications” OR “patient-nurse interaction” OR “patient-nurse interactions”	171
21	“patient-hospital relation” OR “patient-hospital relations” OR “patient-hospital relationship” OR “patient-hospital relationships” OR “patient-hospital communication” OR “patient-hospital communications” OR “patient-hospital interaction” OR “patient-hospital interactions”	10
22	(((((“Patient Participation”[Mesh]) OR “Community Participation”[Mesh]) OR “Patient-Centered Care”[Mesh]) OR “Decision Making”[Mesh]) OR “Hospital-Patient Relations”[Mesh]) OR “Physician-Patient Relations”[Mesh] OR “Culturally competent care”[Mesh]	307.973
23	Or/1-22	465.523
**Block 2**		
24	Migrant OR migrants	18.751
25	Immigrant OR immigrants	24.342
26	“ethnic minority” OR “ethnic minorities” OR “ethnic minority group” OR “ethnic minority groups”	10.547
27	Refugee OR refugees	10.136
28	“asylum seeker” OR “asylum seekers”	1.506
29	Descendant OR descendants	5.445
30	“undocumented immigrant” OR “undocumented immigrants” OR “illegal immigrant” OR “illegal immigrants”	585
31	(((“Emigrants and Immigrants”[Mesh]) OR “Transients and Migrants”[Mesh]) OR “Refugees”[Mesh]) OR “Undocumented Immigrants”[Mesh]	30.643
32	OR/24-31	71.957
**Block 3**		
33	“healthcare service” OR “healthcare services” OR “health care service” OR “health care services”	23.170
	“delivery of healthcare” OR “delivery of health care”	11.650
	“Integrated delivery of healthcare” OR “Integrated delivery of health care”	27
37	((“Health Services”[Mesh]) OR (“Delivery of Health Care”[Mesh] OR “Delivery of Health Care, Integrated”[Mesh]))	2.681.002
38	OR/33-37	2.691.281
39	23 AND 32 AND 38	2.300
	23 AND 32 AND 38 only: English, Swedish, Norwegian, Danish, German	2.197
	**From 2007 onwards**	**1.604**

## References

[cit0001] Adams, K. M., Gardiner, L. D., & Assefi, N. (2004). Healthcare challenges from the developing world: Post-immigration refugee medicine. *BMJ (Clinical Research Ed.)*, 328(7455), 1548–17. 10.1136/bmj.328.7455.1548 PMC437153 15217874

[cit0002] Ahmed, S., Lee, S., Shommu, N., Rumana, N., & Turin, T. (2017). Experiences of communication barriers between physicians and immigrant patients: A systematic review and thematic analysis. *Patient Experience Journal*, 4(1), 122–140. 10.35680/2372-0247.1181

[cit0003] Alegria, M., Falgas-Bague, I., & Fong, H.-F. (2020). Engagement of ethnic minorities in mental health care. *World Psychiatry*, 19(1), 35–36. 10.1002/wps.20695 31922667 PMC6953572

[cit0004] Radl-Karimi, C., Nicolaisen, A., Sodemann, M., Batalden, P., & von Plessen, C. (2018). Coproduction of healthcare service with immigrant patients: protocol of a scoping review. *BMJ Open, 8*(2), 1–7. 10.1136/bmjopen-2017-019519 PMC5829924 29431137

[cit0005] Balachandra, S. K., Carroll, J. K., Fogarty, C. T., & Finigan, E. G. (2009). Family-centered maternity care for deaf refugees: The patient-centered medical home in action. *Families, Systems & Health: The Journal of Collaborative Family HealthCare*, 27(4), 362–367. 10.1037/a0018214 20047358

[cit0006] Balán, I. C., Moyers, T. B., & Lewis-Fernandez, R. (2013). Motivational pharmacotherapy: Combining motivational interviewing and antidepressant therapy to improve treatment adherence. *Psychiatry: Interpersonal and Biological Processes*, 76(3), 203–209. 10.1521/psyc.2013.76.3.203 PMC4507411 23965260

[cit0007] Ballatt, J., Campling, P., & Maloney, C. (2020). *Intelligent kindness. Rehabilitating the welfare state* (2nd ed.). Cambridge University Press.

[cit0008] Baraldi, C., & Gavioli, L. (2013). Mediating patient-centred interaction: The case of doctors’ questions. *Salute e Societa*, 1(1), 94–120. 10.3280/SES2013-001008

[cit0009] Batalden, M., Batalden, P., Margolis, P., Seid, M., Armstrong, G., Opipari-Arrigan, L., & Hartung, H. (2015). Coproduction of healthcare service. *BMJ Quality & Safety*, *25*(7), 1–9. 10.1136/bmjqs-2015-004315 PMC4941163 26376674

[cit0010] Beagan, B. L., & Kumas-Tan, Z. (2009). Approaches to diversity in family medicine: “I have always tried to be colour blind”. *Canadian Family Physician Medecin De Famille Canadien*, 55(8), e21–28.https://pubmed.ncbi.nlm.nih.gov/19675253/19675253 PMC2726109

[cit0011] Benson, J., Haris, T. A., & Saaid, B. (2010). The meaning and the story: Reflecting on a refugee’s experiences of mental health services in Australia. *Mental Health in Family Medicine*, 7(1), 3–8. https://pubmed.ncbi.nlm.nih.gov/22477917; https://www.ncbi.nlm.nih.gov/pmc/articles/PMC2925159/22477917 PMC2925159

[cit0012] Bernard, W. (1976). Immigrants and Refugees: Their Similarities, Differences, and Needs. *International Migration*, 14(4), 267–280. 10.1111/j.1468-2435.1976.tb00947.x 12337494

[cit0013] Bischoff, A., Kurth, E., & Henley, A. (2012). Staying in the middle: A qualitative study of health care interpreters’ perceptions of their work. *Interpreting*, 14(1), 1–22. 10.1075/intp.14.1.01bis

[cit0014] Borkan, J. M., Culhane-Pera, K. A., & Goldman, R. E. (2008). Towards cultural humility in healthcare for culturally diverse Rhode Island. *Medicine and Health, Rhode Island*, 91(12), 361–364. http://ovidsp.ovid.com/ovidweb.cgi?T=JS&PAGE=reference&D=emed10&NEWS=N&AN=354388779 19170310

[cit0015] Bovaird, T. (2007). Beyond Engagement and Participation: User and Community Coproduction of Public Services. *Public Administration Review*, 67(5), 846–860. 10.1111/j.1540-6210.2007.00773.x

[cit0016] Bovaird, T., Flemig, S., Loeffler, E., & Osborne, S. P. (2019). How far have we come with co-production—and what’s next? *Public Money & Management*, 39(4), 229–232. 10.1080/09540962.2019.1592903

[cit0017] Brämberg, E. B., Nyström, M., & Dahlberg, K. (2010). Patient participation: A qualitative study of immigrant women and their experiences. *International Journal of Qualitative Studies on Health and Well-being*, 5(1), 10.3402/qhw.v5i1.4650 PMC2879867 20640027

[cit0018] Brassart, E., Prévost, C., Bétrisey, C., Lemieux, M., & Desmarais, C. (2017). Strategies developed by service providers to enhance treatment engagement by immigrant parents raising a child with a disability. *Journal of Child and Family Studies*, 26(4), 1230–1244. 10.1007/s10826-016-0646-8

[cit0019] Clarke, S. K., Jaffe, J., & Mutch, R. (2019). Overcoming communication barriers in refugee health care. *Pediatric Clinics of North America*, 66(3), 669–686. 10.1016/j.pcl.2019.02.012 31036242

[cit0020] Cochran, D., Saleem, S., Khowaja-Punjwani, S., &Lantos, J. D. (2017). Cross-cultural differences in communication about a dying child. *Pediatrics*, 140(5), 5. 10.1542/peds.2017-0690 29051330

[cit0021] Coulehan, J. L., Platt, F. W., Egener, B., Frankel, R., Lin, C. T., Lown, B., & Salazar, W. H. (2001). “Let me see if i have this right … ”: Words that help build empathy. *Annals of Internal Medicine*, 135(3), 221–227. 10.7326/0003-4819-135-3-200108070-00022 11487497

[cit0022] Coulter, A., & Ellins, J. (2006). *Patient-focused interventions: A review of the evidence*. The Health Foundation.

[cit0023] DeCamp, L. R., Gregory, E., Polk, S., Camacho Chrismer, M., Giusti, F., Thompson, D. A., & Sibinga, E. (2015). A voice and a vote: The Advisory board experiences of Spanish-speaking Latina mothers. *Hispanic Health Care International*, 13(4), 217–226. 10.1891/1540-4153.13.4.217 26671562 PMC4751862

[cit0024] Derose, K. P., Escarce, J. J., & Lurie, N. (2007). Immigrants and health care: Sources of vulnerability. *Health Affairs*, 26(5), 1258–1268. 10.1377/hlthaff.26.5.1258 17848435

[cit0025] Dias, S., Gama, A., Cargaleiro, H., & Martins, M. O. (2012). Health workers’ attitudes toward immigrant patients: A cross-sectional survey in primary health care services. *Human Resources for Health*, 10(1), 14. 10.1186/1478-4491-10-14 22776316 PMC3422994

[cit0026] Doescher, M. P., Saver, B. G., Franks, P., & Fiscella, K. (2000). Racial and ethnic disparities in perceptions of physician style and trust. *Archives of Family Medicine*, 9(10), 1156–1163. 10.1001/archfami.9.10.1156 11115223

[cit0027] Elwyn, G., Nelson, E., Hager, A., & Price, A. (2019). Coproduction: When users define quality. *BMJ Quality & Safety*, *29,* 1–6. 10.1136/bmjqs-2019-009830 PMC7467503 31488570

[cit0028] Fragkos, K. (2016). Reflective practice in healthcare education: An umbrella review. *Education Sciences*, 6(4), 27. 10.3390/educsci6030027

[cit0029] Gonsalves, C. J. (1992). Psychological stages of the refugee process: A model for therapeutic interventions. *Professional Psychology, Research and Practice*, 23(5), 382–389. 10.1037/0735-7028.23.5.382

[cit0030] Greenfield, D., Eljiz, K., & Butler-Henderson, K. (2018). It takes two to Tango: Customization and standardization as colluding logics in healthcare comment on “(re) making the procrustean bed standardization and customization as competing logics in healthcare”. *International Journal of Health Policy and Management*, 7(2), 183–185. 10.15171/ijhpm.2017.77 29524942 PMC5819378

[cit0031] Greenhalgh, T. (1999). Narrative based medicine in an evidence based world. *BMJ*, 318(7179), 323–325. 10.1136/bmj.318.7179.323 9924065 PMC1114786

[cit0032] Greenhalgh, T. (2013). The compassionate organisation. *British Journal of General Practice*, 63(614), 481. 10.3399/bjgp13X671669 PMC3750781 23998822

[cit0033] Gustafsson, K., Norström, E., & Fioretos, I. (2013). The interpreter - a cultural broker. In C. Schäffner, K. Kredens, & Y. Fowler (Eds.), *Interpreting in a changing landscape: Selected papers from critical link 6* (pp. 187–202). John Benjamins Publishing Company.

[cit0034] Hall, E. T. (1981). *Beyond culture*. Anchor Books.

[cit0035] Hardy, C. (2017). Empathizing with patients: The role of interaction and narratives in providing better patient care. *Medicine, Health Care, and Philosophy*, 20(2), 237–248. 10.1007/s11019-016-9746-x 27796726

[cit0036] Hardyman, W., Daunt, K. L., & Kitchener, M. (2015). Value co-creation through patient engagement in health care: A micro-level approach and research agenda. *Public Management Review*, 17(1), 90–107. 10.1080/14719037.2014.881539

[cit0037] Hillen, M. A., De Haes, H., Verdam, M. G. E., & Smets, E. M. A. (2018). Trust and perceptions of physicians’ nonverbal behavior among women with immigrant backgrounds. *Journal of Immigrant and Minority Health*, 20(4), 963–971. 10.1007/s10903-017-0580-x 28391500 PMC6061085

[cit0038] Holland-Hart, D. M., Addis, S. M., Edwards, A., Kenkre, J. E., & Wood, F. (2019). Coproduction and health: Public and clinicians’ perceptions of the barriers and facilitators. *Health Expectations: An International Journal of Public Participation in Health Care and Health Policy*, 22(1), 93–101. 10.1111/hex.12834 30289592 PMC6351407

[cit0039] Hupcey, J. E., Penrod, J., Morse, J. M., & Mitcham, C. (2001). An exploration and advancement of the concept of trust. *Journal of Advanced Nursing*, 36(2), 282–293. 10.1046/j.1365-2648.2001.01970.x 11580804

[cit0040] Innes, A. D., Campion, P. D., & Griffiths, F. E. (2005). Complex consultations and the ‘edge of chaos’. *The British Journal of General Practice : The Journal of the Royal College of General Practitioners*, 55(510), 47–52.https://www.ncbi.nlm.nih.gov/pmc/articles/PMC1266243/15667766 PMC1266243

[cit0041] Jakobsen, M., & Andersen, S. C. (2013). Coproduction and equity in public service delivery. *Public Administration Review*, 73(5), 704–713. 10.1111/puar.12094

[cit0042] Joiner, L., & Lusch, R. (2016). Evolving to a new service-dominant logic for health care. *Innovation and Entrepreneurship in Health*, 3, 25–33. 10.2147/IEH.S93473

[cit0043] Jones, J., Bion, J., Brown, C., Willars, J., Brookes, O., Tarrant, C., … The, P. C. (2020). Reflection in practice: How can patient experience feedback trigger staff reflection in hospital acute care settings? *Health Expectations*, 23(2), 396–404. 10.1111/hex.13010 31858677 PMC7104653

[cit0044] Kaltman, S., Serrano, A., Talisman, N., Magee, M. F., Cabassa, L. J., Pulgar-Vidal, O., & Peraza, D. (2016). Type 2 diabetes and depression: A pilot trial of an integrated self-management intervention for Latino immigrants. *The Diabetes Educator*, 42(1), 87–95. 10.1177/0145721715617536 26590385

[cit0045] King, G., Desmarais, C., Lindsay, S., Pierart, G., & Tetreault, S. (2015). The roles of effective communication and client engagement in delivering culturally sensitive care to immigrant parents of children with disabilities. *Disability and Rehabilitation*, 37(15), 1372–1381. 10.3109/09638288.2014.972580 25323397

[cit0046] Laine, C., & Davidoff, F. (1996). Patient-centered medicine. A professional evolution. *JAMA*, 275(2), 152–156. 10.1001/jama.1996.03530260066035 8531314

[cit0047] Langlois, E. V., Haines, A., Tomson, G., & Ghaffar, A. (2016). Refugees: Towards better access to health-care services. *Lancet (London, England)*, 387(10016), 319–321. 10.1016/S0140-6736(16)00101-X PMC5603273 26842434

[cit0048] Launer, J. (1999). A narrative approach to mental health in general practice. *BMJ*, 318(7176), 117–119. 10.1136/bmj.318.7176.117 9880290 PMC1114579

[cit0049] Lee, S. K., Thompson, S. C., & Amorin-Woods, D. (2009). One service, many voices: Enhancing consumer participation in a primary health service for multicultural women. *Quality in Primary Care*, 17(1), 63–69. 19281676

[cit0050] Loeffler, E., Power, G., Bovaird, T., & Hine-Hughes, F. (2013). *Co-production of health and wellbeing in Scotland*. Governance International.

[cit0051] Mead, E. L., Doorenbos, A. Z., Javid, S. H., Haozous, E. A., Alvord, L. A., Flum, D. R., & Morris, A. M. (2013). Shared decision-making for cancer care among racial and ethnic minorities: A systematic review. *American Journal of Public Health*, 103(12), e15–29. 10.2105/ajph.2013.301631 PMC3828995 24134353

[cit0052] Mendenhall, T. J., Kelleher, M. T., Baird, M. A., & Doherty, W.J. (2008). Overcoming Depression in a Strange Land: A Hmong Woman’s Journey in the World of Western Medicine. In: Kessler, R., & Stafford, D. (eds) Collaborative Medicine Case Studies. Springer. https://doi-org.proxy1-bib.sdu.dk/10.1007/978-0-387-76894-6_27Mendenhall, T. J., Kelleher, M. T., Baird, M. A., & Doherty, W. J. (2008). Overcoming depression in a strange land: A Hmong woman’s journey in the world of Western medicine. *Collaborative Medicine Case Studies: Evidence in Practice*, 327–340. 10.1007/978-0-387-76894-6_27

[cit0053] Murdoch-Eaton, D., & Sandars, J. (2014). Reflection: Moving from a mandatory ritual to meaningful professional development. *Archives of Disease in Childhood*, 99(3), 279–283. 10.1136/archdischild-2013-303948 23975720

[cit0054] Naldemirci, O., Lydahl, D., Britten, N., Elam, M., Moore, L., & Wolf, A. (2018). Tenacious assumptions of person-centred care? Exploring tensions and variations in practice. *Health (London)*, 22(1), 54–71. 10.1177/1363459316677627 27879342

[cit0055] Needham, C., & Carr, S. (2009). *Co-production: An emerging evidence base for adult social care transformation*. Social Care Institute for Excellence.

[cit0056] O’Toole, J. K., Alvarado-Little, W., & Ledford, C. J. W. (2019). Communication with diverse patients: Addressing culture and language. *Pediatric Clinics of North America*, 66(4), 791–804. 10.1016/j.pcl.2019.03.006 31230623

[cit0057] Ocloo, J., & Matthews, R. (2016). From tokenism to empowerment: Progressing patient and public involvement in healthcare improvement. *BMJ Quality & Safety*, 25(8), 626–632. 10.1136/bmjqs-2015-004839 PMC4975844 26993640

[cit0058] Osborne, S. P., Radnor, Z., & Strokosch, K. (2016). Co-production and the co-creation of value in public services: A suitable case for treatment? *Public Management Review*, 18(5), 639–653. 10.1080/14719037.2015.1111927

[cit0059] Osborne, S. P., & Strokosch, K. (2013). It takes two to Tango? Understanding the co-production of public services by integrating the services management and public administration perspectives: It takes two to Tango? *British Journal of Management*, 24(1), 31–47. 10.1111/1467-8551.12010

[cit0060] Oxford English Dictionary, (Ed.). (2020). *Oxford Dictionaries*. Oxford University Press.

[cit0061] Pellegrini, C. A. (2017). Trust: The Keystone of the patient-physician relationship. *Journal of the American College of Surgeons*, 224(2), 95–102. 10.1016/j.jamcollsurg.2016.10.032 27773776

[cit0062] Perreira, K. M., & Ornelas, I. (2013). Painful passages: Traumatic experiences and post-traumatic stress among immigrant Latino adolescents and their primary caregivers. *International Migration Review*, 47(4), 976–1005. 10.1111/imre.12050 PMC3875301 24385676

[cit0063] Polo, A. J., Alegría, M., & Sirkin, J. T. (2012). Increasing the engagement of Latinos in service through community-derived programs: The right question project–mental health. *Professional Psychology, Research and Practice*, 43(3), 208–216. 10.1037/a0027730

[cit0064] Prasad, S. J., Nair, P., Gadhvi, K., Barai, I., Danish, H. S., & Philip, A. B. (2016). Cultural humility: Treating the patient, not the illness. *Medical Education Online*, 21(1), 30908. 10.3402/meo.v21.30908 26847853 PMC4742464

[cit0065] Priebe, S., Sandhu, S., Dias, S., Gaddini, A., Greacen, T., Ioannidis, E., … Bogic, M. (2011). Good practice in health care for migrants: Views and experiences of care professionals in 16 European countries. *BMC Public Health*, 11(1), 187. 10.1186/1471-2458-11-187 21439059 PMC3071322

[cit0066] Ranjan, P., Kumari, A., & Chakrawarty, A. (2015). How can doctors improve their communication skills? *Journal of Clinical and Diagnostic Research : JCDR*, 9(3), Je01–04. 10.7860/jcdr/2015/12072.5712 PMC4413084 25954636

[cit0067] Riva, S., Monti, M., Iannello, P., Pravettoni, G., Schulz, P. J., & Antonietti, A. (2014). A preliminary mixed-method investigation of trust and hidden signals in medical consultations. *PloS One*, 9(3), e90941. 10.1371/journal.pone.0090941 24618683 PMC3949702

[cit0068] Tricco, A. C., Lillie, E., Zarin, W., O’Brien, K., Colquhoun, H., Kastner, M., … Straus, S. E. (2016). A scoping review on the conduct and reporting of scoping reviews. *BMC Medical Research Methodology*, 16(1), 15. 10.1186/s12874-016-0116-4 26857112 PMC4746911

[cit0069] Tricco, A. C., Lillie, E., Zarin, W., O’Brien, K., Colquhoun, H., Levac, D., … Straus, S. E. (2018). PRISMA extension for scoping reviews (PRISMA-ScR): Checklist and explanation. *Annals of Internal Medicine*, 169(7), 467–473. 10.7326/m18-0850 30178033

[cit0070] United Nations, Department of Economic and Social Affairs, Population Division. (2019). International Migration 2019: Report (ST/ESA/SER.A/438). United Nations.

[cit0071] Weick, K. E. (1995). *Sensemaking in organizations*. Sage Publications.

[cit0072] Whittal, A., Hanke, K., & Lippke, S. (2017). Investigating acculturation orientations of patients with an immigration background and doctors in Canada: Implications for medical advice adherence. *Quality of Life Research*, 26(5), 1223–1232. 10.1007/s11136-016-1438-8 27761682

[cit0073] Wilson, S., Morse, J. M., & Penrod, J. (1998). Developing reciprocal trust in the caregiving relationship. *Qualitative Health Research*, 8(4), 446–465. 10.1177/104973239800800402 10558339

